# Effect of probiotics as a complement to non-surgical periodontal therapy in chronic periodontitis: a systematic review

**DOI:** 10.4317/medoral.23147

**Published:** 2020-01-01

**Authors:** Anna Vives-Soler, Eduardo Chimenos-Küstner

**Affiliations:** 1 Unity of Oral Medicine, Department of Odontostomatology, University of Barcelona

## Abstract

**Background:**

To improve the results of the classic periodontal treatment, probiotics have been suggested recently to decrease the number of bacteria and the expression of mediators of inflammation. This systematic review aimed to assess the literature for the effectiveness of different probiotic strains as adjuvants to non-surgical periodontal therapy.

**Material and Methods:**

The electronic database of MEDLINE (via Pubmed) was searched up to December 2017 for randomised clinical trials in English comparing non-surgical periodontal treatment and probiotics versus periodontal treatment and placebo. The primary outcome investigated was reduction in pocket probing depth. Secondary outcomes were bleeding on probing, plaque index reduction and bacteria counts.

**Results:**

Nine trials were included. A narrative data synthesis did not result in any major improvement of overall pocket probing depth but moderate pockets from 4 to 6 mm showed larger reductions in study groups, which could decrease the need for surgery. Sites with bleeding on probing and presence of plaque decreased after treatment. For periimplant mucositis, there was a small tendency to better results in the study group.

**Conclusions:**

With the available data, it is concluded that probiotics may provide an additional benefit to manual debridement in chronic periodontitis. More studies are required on dose, route of administration and strains of probiotics used.

** Key words:**Periodontics, chronic periodontitis, probiotics, periodontal treatment, lactobacillus, streptococcus.

## Introduction

Periodontal disease is defined as an inflammation of the supporting tissues of the teeth, leading to loss of bone and periodontal ligament. Some oral bacteria species, organised in biofilms, are considered one of the main etiologic factors, triggering a local destructive inflammatory reaction that creates periodontal pockets. Conventional periodontal treatment is mainly comprised of mechanical debridement, since both the disruption of the biofilm and the restoration of a biologically accepTable root surface are important in periodontitis progression (1). Thus, a probing pocket depth (PPD) reduction is normally taken as an indicator of treatment success and means good control over the inflammatory process (2).

To improve the results of the classic periodontal treatment, some alternative therapies have been suggested recently to decrease the number of bacteria and the expression of mediators of inflammation. Preliminary studies show promising results using probiotics for oral diseases such as caries and candidiasis. Probiotics are living microorganisms which, when administered in adequate amounts, confer a health benefit for the host (3). However, there is *in vitro* and animal evidence that probiotic preparations comprised of dead cells and their metabolites can also exert a biological response (4). Probiotics’ exact mechanism of action in the oral cavity isn’t fully understood: there may be a direct interaction with the dental plaque, disrupting the biofilm owing to their antimicrobial products and competitive adhesion, and an indirect action as well, modulating the host’s immune response.

The aim of this study was to review the adjuvant effects of different probiotic strains together with root scaling on the clinical outcomes of chronic periodontitis (CP) patients.

## Material and Methods

- Study design

This was a systematic review carried out using the PRISMA Statement as a reporting guide (5).

- Eligibility criteria

Only randomised clinical trials in English were considered. Eligible trials had to compare combined manual therapy and probiotics, or manual therapy and placebo. A reduction in probing pocket depth (PPD) was the main outcome measure. Secondary outcome measures were: bleeding on probing (BOP), plaque index (PI), and bacteria counting. Non-randomised clinical trials, studies conducted in animals and those including patients with a healthy periodontium or experimental gingivitis were excluded.

- Search strategy and study selection

We conducted an electronic search in Medline (via PUBMED) with controlled vocabulary (MeSH) using the following keywords: “Probiotics” and “Periodontal Disease”, with no date restrictions. Last search was conducted in December of 2017. Relevant articles were selected and assessed, and cross-referencing from these sources was used to identify additional trials. The trials identified were screened by two independent reviewers to determine whether they met or not the inclusion criteria. Any disagreement was resolved after discussion.

- Data collection process

Studies that fulfilled the inclusion criteria were processed for quality assessment. Data were recorded according to the criteria outlined by the Cochrane Collaboration, as done by other authors (6).

- Data items

The following items were recorded: year of publication, study design and duration; size of sample and stage of the disease; test and control interventions, including probiotic strains and any pre-treatment, vehicle, dose, frequency and length of consumption. We recorded the following outcomes: reduction in probing pocket depth, reduction of sites with bleeding on probing, reduction of plaque index scores, changes in bacteria concentration at any site of the oral cavity.

- Risk of bias in individual studies

The risk of bias of the individual studies was assessed according to the Cochrane Handbook for Systematic Reviews of Interventions (6) (high, unclear or low) according to the assessment of the following items: generation of allocation sequences, allocation concealment, blinding of participants and personnel, blinding of outcome assessment, incomplete outcome data, presence of biases in outcome reporting and other sources of bias that could influence the study’s validity. Studies were defined as low risk of bias if all criteria were met. When missing one of these criteria, the study was classified as moderate, and it resulted in a high potential risk when two or more were missing.

- Data analysis

Data extraction revealed considerable heterogeneity of the included studies so that a meta-analysis could not be carried out. Instead of that, data were pooled into Tables and Figures and a descriptive summary was generated to report on study characteristics, results and dissimilarities.

## Results

- Study selection

**Figure 1:**
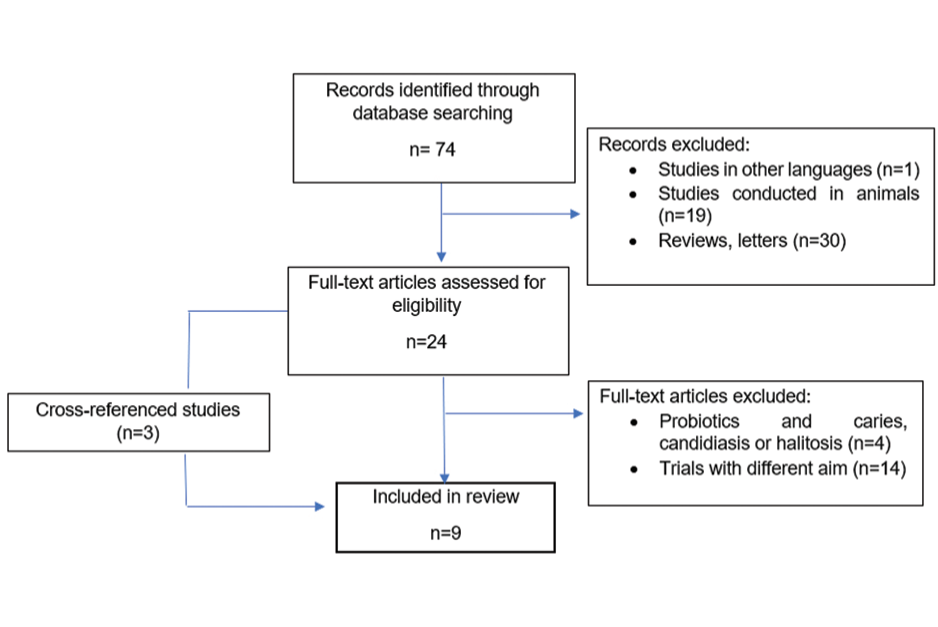
Flowchart of the process of study selection.

From 74 identified studies, six papers fulfilled the eligibility criteria, and three more studies were included from references. The flowchart of the retrieved studies is presented in Fig. 1.

- Characteristics of studies included in the review

Nine randomised control trials published between 2013 and 2016 were included. (3,7-14) Eight trials (3,7-13) used a parallel-arm design, and one of the studies (14) used a split-mouth design. Considerable heterogeneity was observed in all trials with respect to evaluation period, age of participants and case definition (as some used Van der Welden’s (15) definition whereas others used Armitage’s (16)). The main characteristics of the studies are displayed in Table 1.

**Table 1:**
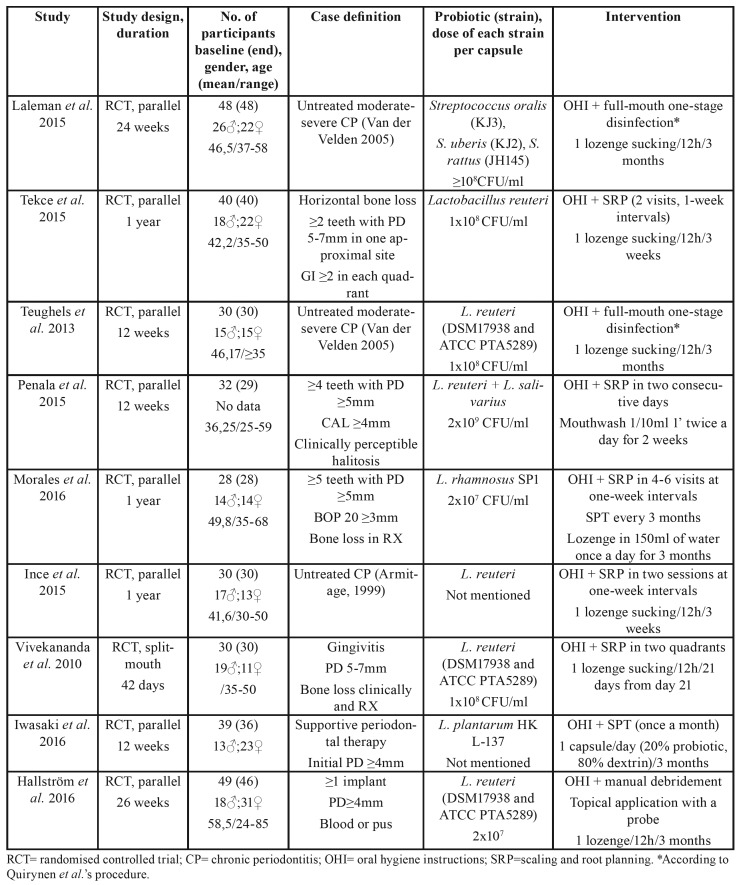
Main characteristics of the included studies.

Subject characteristics

Sample sizes ranged between 28 and 49 participants, with an overall baseline sample size of 326 participants. A sum of nine patients were lost to follow up, in three studies (8,11,13). The gender distribution was mostly reported for the total sample, except in the study of Penala (8). Two studies (12,13) allowed for current smokers, whereas two exclude patients with a history of smoking (8,10), three exclude current smokers only (3,7,9) and one doesn’t mention this criterion (11). The effect of smoking status on the outcome parameters wasn’t subanalysed. Good general health was an inclusion criterion for seven studies (3,7-10,12,14). Hallström *et al*. (13) excluded patients with a history of Diabetes mellitus only, and Iwasaki *et al*. (11) doesn’t mention anything about the baseline health status.

Case definition

Specific selected patients diagnosed with untreated chronic periodontitis were included in seven of the studies (3,7-10,12,14). One of these studies (8) used the presence of clinically perceptible halitosis estimated on an organoleptic score as an additional inclusion criterion. One study (11) took patients undergoing a supportive periodontal therapy (SPT) with an initial PD of ≥4mm. Patients with periimplantitis in at least one implant (a PD≥4mm and presence of pus or bleeding) were included in the study of Hallström *et al*. (13) As the definition of chronic periodontitis differs between all studies, it is shown in Table 1.

Probiotics and intervention procedures

The strains of probiotics used were: L. reuteri (7,9,10,13,14), L. reuteri plus, L. salivarius (8), **Streptococcus* oralis*, *S. uberis* and S. ratus (3), *L. rhamnosus* (12) and L. plantarum (11). All patients were given oral hygiene instructions before treatment, continued by a scaling and root planning in two consecutive days (3,7,8) according to Quirynen’s procedure (17) two sessions at one-week intervals (9,10) or four to six sessions at one-week intervals (12). Only one study (12) performed supportive periodontal therapy (SPT) afterwards, every three months during the evaluations. In one of the studies (11) all patients had undergone a periodontal treatment before the start of the trial and were under an SPT program every month. Probiotic administration was started at the onset of the initial therapy in four studies (9-11,13), after the last session of scaling in four studies (3,7,8,12) and 21 days after treatment in one study (14). The administration routes were as follows: taking capsules (11), Tablet dissolution in mouth (3,7,9,10,13,14), Tablet dissolution in 150ml of water (12), subgingival irrigation plus mouthwash (8) and topical application of ointment (13). Treatment duration ranged from 3 weeks to 3 months (Table 1).

Side effects & industry funding

All studies evaluated the presence of side effects. No adverse events were reported during the follow-up period. Study funding and supporting grants were mentioned in eight studies, (3,7-10,12-14) and in one study (11) it was not mentioned, and they stated to prepare their own probiotic product.

- Risk of bias assessment of selected studies

The estimated potential risk of bias was considered to be “low” in 6 studies (3,7,9-12), moderate in two (8,13) and high in Vivekananda’s (14) study.

- Study outcomes

Pocket probing depth (PPD)

Overall mean PPD at the beginning and at different time points is displayed in Table 2.

**Table 2:**
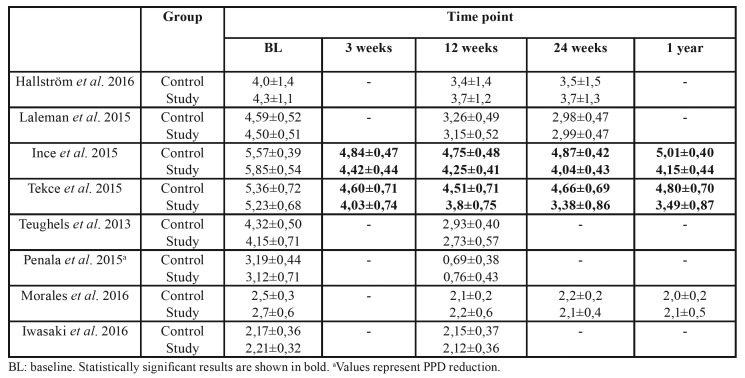
Intergroup comparison of overall PPD (mm) reported at different time points (mean ± SD).

Mean baseline PPD ranges between 2,17±0,36 and 5,57±0,39 mm in the control group and 2,21±0,32 and 5,85±0,54mm for the study group. At 12 weeks, values range from 0,69±0,38 to 4,75±0,48 mm and from 0,76±0,43 to 4,25±0,41 mm for the control and study groups respectively. Statistically significant differences in favour of the study group were seen in two studies at all time points (*p*<0,001) (9,10).

Four out of five studies that evaluated PPD reduction in moderate pockets (4-6 mm) found statistically significant differences between the control and study groups, with greater reductions for the study group. (7-9,11) One study (3) found a greater reduction in the control group, which was not significant (Table 3).

**Table 3:**
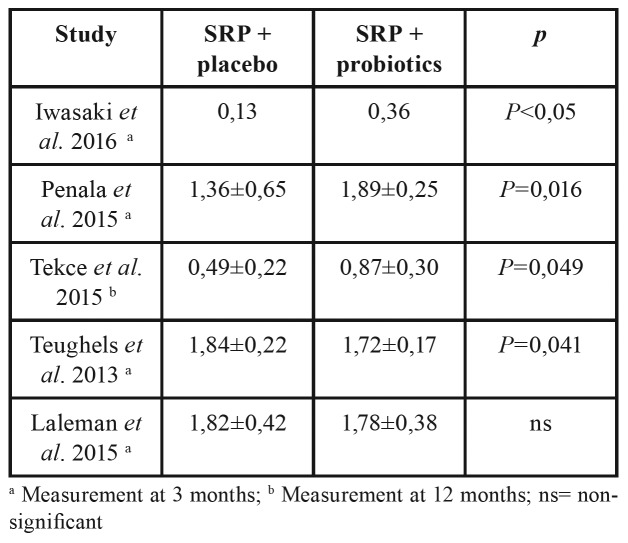
PPD reduction (mm) in moderate pockets (4-6mm) for each group at the end of the study.

Bleeding on probing (BOP)

**Figure 2:**
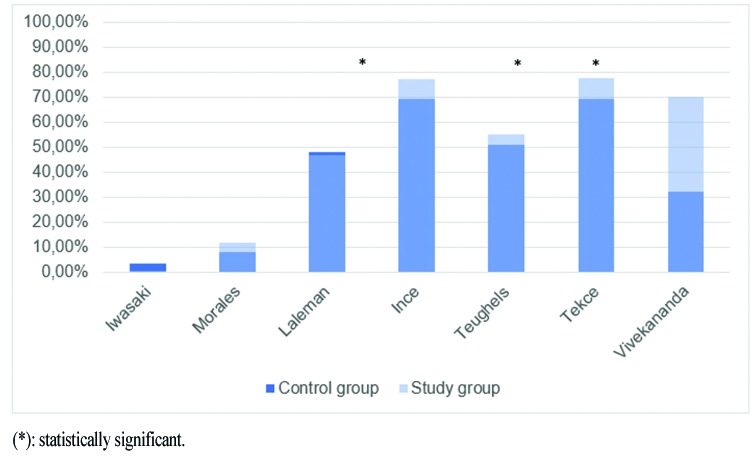
Reduction in bleeding on probing (% of sites) for chronic periodontitis.

The reduction in % of sites with BOP ranges between 3,70-59,5% and 0,70-77,85% for the control and study groups respectively. For chronic periodontitis, there is greater reduction in % of sites with BOP at all time points with the use of probiotics in five studies (7,9,10,12,14). Despite this tendency, only three trials (9,10,14) found statistically significant differences when compared to the control group (*p*<0,005 (9), *p*<0,05 (10) and *p*<0,001 (14)) (Fig. 2). Better results with the use of probiotics have been reported as well when using the bleeding index of Saxton, with a mean reduction of 1,34 and 1,08 (*p*<0,002) (8). Two studies reported a larger reduction in the control group which wasn’t statistically significant: 3,7% vs 0,7% (11) and 48±19% vs 47±17% (3) in control and study groups respectively. For periimplant mucositis, BOP sites were reduced from 54 to 14% in the study group and from 58 to 17% of sites in the control group, which was a statistically significant reduction from baseline but not between groups.

Plaque index (PI)

**Figure 3:**
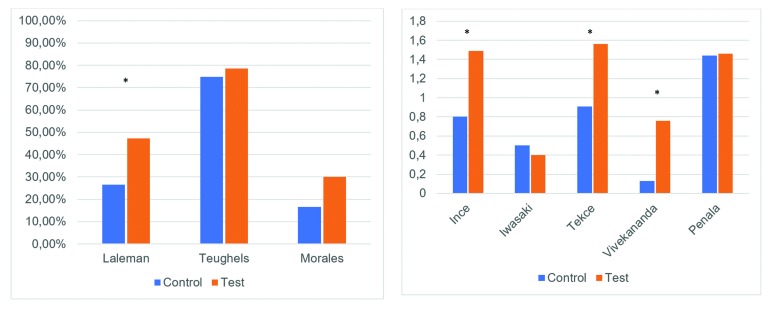
Reduction of % of sites with plaque accumulation and reduction of mean plaque index (Löe and Silness plaque index).

Four trials (3,7,12,13) used a dichotomous measurement for supragingival plaque accumulation and reported % of positive sites to plaque presence. Five trials (8-11,14) report the mean of Löe and Silness’ index (18). Larger reductions in plaque index are seen for study group in seven studies (3,7-10,12,14) but three of them (7,8,12) didn’t find intergroup differences (Fig. 3).

Biochemical and microbiological parameters

There is a significant reduction in red complex microorganisms at 1 month, which was higher for the study group, according to the BANA test (8). Between-group comparisons show a higher reduction of **Aggregatibacter* actinomycetencomitans*, **Porphyromonas* gingivalis* and *Prevotella* intermedia in subgingival plaque at 6-week evaluation for the study group (*p*<0,005, *p*<0,005 and *p*<0,05 respectively) (14), less concentration of *Prevotella* intermedia in saliva samples (*p*<0,02) (3) and of **Porphyromonas* gingivalis* in saliva, supragingival and subgingival plaque (7). Halitosis score, assessed subjectively on a scale of 0 to 5, showed better results with the adjunctive use of probiotics (from 4±0,93 to 0,4±0,51 in the test group and from 4,43±0,51 to 1,21±0,89 in the control group) (8). One study (10) reported a significant decrease in MMP-8 and significant increase in TIMP-1 concentrations that was superior for all time intervals in the study group except day 360.

## Discussion

Chronic periodontitis is a multifactorial disease that arises from the interaction between three key factors: bacteria, the host’s immune response and the environment. Initial therapy consists of a manual debridement of the periodontal pocket and oral hygiene instructions, in order to decrease the number of pathogenic bacteria. However, this shift to a less pathogenic microbiota is only temporary, even when combined with antiseptics or antibiotics (7). Probiotics seem to confer a beneficial effect to many infectious diseases, including those of the oral cavity. A direct interaction with pathogenic flora, a modulation of the immune response and the synthesis of antimicrobial products seem to be some of their mechanisms. Most studies conducted in humans use *Lactobacillus* and *Bifidobacterium spp*, even though other species such as* Bacillus spp* have shown promising results in experimental periodontitis in animals through the production of spores (19). *Lactobacillus* reuteri forms reuterin (3-hydroxipropionaldehyd), which induces oxidative stress in cells (9). Other suggested effects are: ability to compete with pathogens for epithelial cell adhesion (10), a reduction in the production of proinflammatory cytokines (TNF-α, IL-1β, IL-17), (20) a reduction in MMP-8 expression, which is the main collagenase involved in chronic periodontitis, and an increase of TIMP-1, which is a modulating factor of MMP activity. (10) On the other hand, **Streptococcus* sanguinis* and **Streptococcus* uberis* form hydrogen peroxide, a product that inhibits the growth of **Aggregatibacter* actinomycetencomitans* (3). Such biological interactions could have a beneficial impact in clinical parameters of patients with chronic periodontitis.

No additional benefit is seen in terms of mean PPD except for the studies of Tekce *et al*. (9) and Ince *et al*. (10). These studies have deeper baseline PPD and this could have influenced the greater differences between groups. Nevertheless, subanalyses show that different strains of *Lactobacillus* seem to decrease PPD in moderate periodontal pockets (4-6 mm) when compared to the use of placebo + SRP (7-9,11). This effect is not seen in the study of Laleman *et al*. (3) with the use of **Streptococcus* spp* even though the inclusion criteria and intervention were identical to Teughels *et al*.’s (7) who used of L. reuteri. This could mean data obtained *in vitro* and in animal studies doesn’t necessarily need to show beneficial results in humans, and not all strains of probiotics might be effective. Interestingly, two trials (9,11) have less significant reductions than other authors. In the study of Tekce *et al*. (9), measurements took place at 360 days and without any periodontal support therapy. It could be possible that the beneficial effects of probiotics on moderate pockets were temporary and did not last for more than 3 months. In the study of Iwasaki *et al*. (11) mean values reported were not for mean PPD of pockets with 4-6mm but for PPD of teeth with pockets with 4-6mm and that is why the baseline mean wasn’t over 3,08±0,34 mm for any group, compared with the other studies. Also, their patients were under a supportive periodontal therapy, meaning baseline periodontal pockets were not deep and probiotics might not be beneficial in such cases.

Statistically significant differences are seen in intergroup comparisons for BOP in favour of the use of *Lactobacillus* spp, but this was not seen after the use of **Streptococcus* spp* (3). Iwasaki *et al*. (11) found no significant differences in this clinical parameter between the two groups (3,70 and 0,7% reduction for the control and study group respectively). Interestingly, their patients were under a SPT program, which includes a reinforcement of oral hygiene instructions, and this may have influenced such low bleeding throughout the study. Another study to perform SPT after the treatment was that carried out by Morales *et al*. (12). Similarly, the reduction in BOP was 8,30% for the control group and 11,70% for the study group. Tekce *et al*. (9) and Ince *et al*. (10) measure BOP at one year after 3-week consumption of L. reuteri lozenges with no additional TPS and obtain a reduction of 69,6% and 50,95% in the control group and 77,85% and 77,3% in the study group respectively. Teughels *et al*. (7) showed less BOP on the study group but didn’t obtain statistically significant differences at 3-month evaluation despite using a similar methodology than the aforementioned studies. Also, administration routes other than lozenge dissolution have shown promising results according to Penala *et al*. (8) whose patients were told to mouthwash with a L. reuteri and L. salivarius product.

Microbiological and chemical effects are not reported in all trials. Vivekananda (14) was the first author to suggest L. reuteri’s antimicrobial effect in short-term measurements in a split-mouth study, reporting that probiotic lozenges either alone or in combination with SRP decreased the counts of subgingival A. actinomycetencomitans, **P. gingivalis** and *P. intermedia*. Similar results for subgingival plaque were obtained by Tekce *et al*. (9) who reported larger reductions in obligate anaerobes at days 21, 90 and 180, but showed no difference between groups at day 360, suggesting the beneficial effects of probiotics are temporary. According to Teughels *et al*. (7), larger reductions in **P. gingivalis** were found in subgingival, supragingival and saliva samples over the 12-week period, as well as a reduction in *P. intermedia* in supragingival (*p*=0,074) and saliva (*p*<0,05) samples. On the other hand, **Streptococcus* spp*. antimicrobial effect is not clear as Laleman *et al*. (3) found a significant intergroup difference only for *P. intermedia* in saliva at week 12 (*p*=0,02) but did not find any difference for other anaerobe microorganisms, and no further investigation on the presence of the probiotic subgingivally was conducted. Little is known about the subgingival retention of the delivered probiotic as only the paper of Tekce *et al*. (9) considers this parameter. In his study, L. reuteri was detected in 6 and 11 patients the day 21 and 90 respectively, but the levels decreased by the end of the evaluation period. This indicates that probiotic colonization isn’t immediate, is only temporary and isn’t seen in all patients, what could explain differences in microbiological differences between studies.

When analysing the effect of probiotics in the management of periimplant mucositis, Hallström *et al*. (13) performed a manual debridement combined with professional topical application of a L. reuteri gel and prescription of L. reuteri lozenges twice a day for three months. They reported a general improvement in both the test and placebo groups, with a change in PPD at the implant’s deepest site of 0,7-1,2 mm in both groups. Bleeding on probing was reduced in 40 and 41% in study and control groups respectively. They found a tendency to greater reduction in IL-1RA, IL-8, CCL5, TNF-α and GM-CSF in the crevicular fluid, but intergroup comparisons were not statistically significant for any parameter.

Conclusion

Probiotics may provide a safe additional benefit to manual debridement in clinical and biochemical parameters of chronic periodontitis. Nevertheless, more studies are required with larger cohorts on dose, route of administration and strains of probiotics used.
